# Preparation of Stable POSS-Based Superhydrophobic Textiles Using Thiol–Ene Click Chemistry

**DOI:** 10.3390/polym14071426

**Published:** 2022-03-31

**Authors:** Baoliang Wang, Lili Xing, Tieling Xing, Guoqiang Chen

**Affiliations:** College of Textile and Clothing Engineering, Soochow University, Suzhou 215000, China; 18351287783@163.com (B.W.); 13140877009@163.com (L.X.); xingtieling@suda.edu.cn (T.X.)

**Keywords:** dopamine, click chemistry, POSS, oil–water separation

## Abstract

In this study, a superhydrophobic fabric was synthesized by modifying the fiber’s surface with dopamine-containing hydroxyl functional groups. Furthermore, we introduced mercapto-based functional groups by the hydrolysis of mercaptopropylmethyldimethoxysilane (MPMDS) and finally grafted POSS and mercaptans using a thiol–ene click reaction. These processes generated a superhydrophobic fabric with a static contact and a sliding angle of 162° and 8°, respectively. The superhydrophobic fabric’s compact and regular micro-nano rough structure based on POSS and mercaptans provides stable fastness and durability, as well as high resistance to organic solvents, acid–base environments, mechanical abrasion, UV rays, and washing. Moreover, it can be used for self-cleaning and oil–water separation, and it has a wide range of applications in the coating industry.

## 1. Introduction

After having undergone an extended evolutionary process, organisms demonstrate various wetting phenomena, including the self-cleaning ability of lotus leaves, the high adhesion and super-hydrophobicity of rose petals, and the anti-fogging properties of an insect’s compound eyes. These unique infiltration phenomena have attracted the attention of the scientific community. Numerous studies have focused on this unique surface with a contact angle of >150° and a rolling angle of <10° [1,2,3,4,5]. Various methods for preparing superhydrophobic surfaces have been developed via extensive research, including the sol-gel [6,7], layer-by-layer self-assembly [8,9], and chemical vapor deposition methods [10,11]. Because of the superhydrophobic surface’s unique wettability, it can be used in self-cleaning [12], anti-fog [13,14] and anti-icing [15], anti-corrosion [16], oil–water separation [17], and microfluidic transportation. Mohseni et al. developed a non-fluorinated and simple spraying treatment method for manufacturing durable but comfortable superhydrophobic textiles [18]. However, many issues continue to limit the widespread application of superhydrophobic products at the moment. However, at present, many superhydrophobic coatings are very fragile due to the deviation of fastness and stability and are easily affected by harsh environmental conditions to reduce or lose their superhydrophobic function. This is due to the poor bonding force between the micro-nano-structure of the superhydrophobic surface and the substrate surface, so it is very necessary to improve the fastness and durability of superhydrophobic performance.

The primary component of the viscous protein secreted by the mollusk marine mussel is polydopamine [19,20]. It exhibits super adhesion, which is primarily because of the adjacent polydopamine, hydroquinone, and amine functional groups. Polydopamine has quickly established itself as a significant part in various fields such as medicine [21], sewage treatment [22], and energy materials [23], because of its excellent biocompatibility, hydrophilicity, biodegradability, nontoxicity, and nonpollution properties. In a one-step polymerization and co-deposition reaction triggered by dopamine, Wang et al. used dopamine/ether ammonium sulfate glue to coat the surface of the ultrafiltration membrane. The coating imparts the membrane with a high degree of hydrophilicity [24]. Lu et al. prepared a superhydrophobic magnetic polyurethane sponge by covalent deposition of polydopamine and magnetic metal particles [25]. By oxidation, dopamine self-polymerizes to form a polydopamine coating using a cross-linked network structure. The coating’s strong and universal adhesion is attributed to the diverse interactions and abundance of active sites between it and the substrate. While research into the adhesion mechanism of polydopamine is incomplete, this does not preclude its widespread use in material surface modification, functional coating construction, and composite material preparation.

Recently, POSS has been extensively employed to prepare superhydrophobic surfaces as a developing material [26,27]. As per the self-assembly behavior of POSS-based hybrid polymers, because the inorganic part of POSS and the organic host are incompatible in the polymer, POSS will inevitably accumulate in the organic host to reduce the energy of the system. The polymer’s ability to aggregate on the surface affects its surface morphology. The morphology of protruding particles can significantly increase the substrate’s surface roughness, thereby increasing the substrate’s hydrophobicity. POSS is used to prepare superhydrophobic surfaces in one of three ways. The first method is that POSS is mixed with a hydrophobic polymer and then treated on the surface of the substrate by the dip-coating method to obtain a superhydrophobic effect. The second method introduces POSS as a monomer via chemical covalent bonds into various polymerization systems to obtain different polymer-containing POSS. The reaction is performed using different methods such as free radical polymerization, ATRP, and light-curing, and then applied to the substrate via dip coating, spin coating, spraying, electrostatic spinning, and different ways to create a superhydrophobic and oleophobic surface. The third method directly uses POSS to modify carbon materials such as carbon nanotubes [28,29] and graphene [30]. Both can be used to alter the material’s wettability. POSS materials are used to modify fibers to prepare superhydrophobic textiles; however, this process is tedious. It primarily makes use of POSS’s nano-size, low surface energy, and modifiable properties.

The outstanding advantage of mercapto–olefin click chemistry is that it is simple and fast, and through a series of links between small unit molecules, a polymer with a relatively large molecular weight can be rapidly synthesized. It also has the following advantages: the reaction materials are easily available, the reaction conditions are not harsh, and the grafting rate is high [31,32,33]. Scientists have applied thiol–ene click chemistry to post-functionalization on the surface of materials, and the reaction is simple and efficient, which greatly improves the properties of materials. Zhou et al. [34] synthesized durable superhydrophobic cellulose nanocrystals by grafting polycarboxylate diol based on the principle of click chemistry. Therefore, thiol–ene click chemistry has great research significance in the re-modification and post-functionalization of materials.

In this study, we create a secondary reaction platform by coating polydopamine on the fiber surface, introduce abundant hydroxyl functional groups as a carrier for mercaptosilane hydrolysis, and finally graft POSS and mercaptans via thiol–ene click chemistry. A superhydrophobic textile is prepared on the fiber’s surface. The resulting superhydrophobic fabric exhibits excellent resistance to multiple physical and chemical stresses, including acid–base environments, ultraviolet radiation, mechanical abrasion, organic solvents, and soaping. Moreover, it possesses excellent oil–water separation and self-cleaning performance.

## 2. Experimental

### 2.1. Materials

The nylon 56/cotton-interwoven fabric was purchased from Jiangsu Lianfa Textile Co., Ltd. (Nantong, China) (containing 50% nylon 56 fiber and 50% cotton fiber). Dopamine hydrochloride (98.5%) (DA) (AR) was purchased from Shanghai Yuanye Biotechnology Co., Ltd. (Shanghai, China). Tris(hydroxymethyl)aminomethane and hexadecyl mercaptan were purchased from Shanghai Jingchun Biochemical Technology Co., Ltd. Mercaptopropylmethyldimethoxysilane (97%) (MPMDS) was purchased from Shanghai Saen Chemical Technology Co., Ltd. (Shanghai, China). Eight vinyl-POSS were purchased from Shanghai Aladdin Biochemical Technology Co., Ltd. (Shanghai, China). Tris Hydrochloride was purchased from Suzhou Great Pharmaceutical Technology Co., Ltd. (Suzhou, China). 2,2-Dimethoxy-2-phenylacetophenone (DMPA), absolute ethanol, ethyl acetate, oil red O, methyl blue, reactive blue 4 dye, dichloromethane, carbon tetrachloride, and ethyl acetate were purchased from China Sinopharm Chemical Reagent Co., Ltd. (Shanghai, China).

### 2.2. Preparation of Dopamine-Modified Fabric

Dopamine (0.3 g) was dissolved in an Erlenmeyer flask-containing deionized water (150 mL), the pH was adjusted to 8.5 using Tris and Tris-HCl, and the fabric (5 cm × 5 cm) was placed among them. The flask was kept unsealed and shaken in an oscillating water bath at 45 °C for 24 h. The fabric was removed and ultrasonically vibrated with absolute ethanol to remove the weakly bound polydopamine before drying in an oven at 60 °C for 0.5 h to obtain a dopamine-modified fabric.

### 2.3. Preparation of Mercapto-Modified Fabric

In a dye cylinder, insert the dopamine-modified fabric, add 0.3 mL of mercaptopropyl methyl dimethoxy silane, and allow the reaction to proceed for 1 h at 90 °C in an infrared dyeing machine. Remove the cloth sample, place it in an Erlenmeyer flask, clean it with 100 mL of absolute ethanol, oscillate it in an oscillating sample machine at 25 °C for 1 h, remove, wash with deionized water, and dry in a vacuum at 140 °C.

### 2.4. Preparation of Superhydrophobic Fabric

The mercapto-modified fabric (5 cm × 5 cm) was immersed in 150 mL of ethyl acetate solution. The solution was then treated with hexadecyl mercaptan (0.6 g), octavinyl-POSS (0.2 g), and DMPA (0.25 g). The reaction system was sealed and irradiated for 30 min with a UV lamp (250 W, λ = 365 nm) light source, with the fabric 15 cm away from the light source. Furthermore, the fabric was turned over 15 min after the reaction began. The modified fabric was shaken and washed with absolute ethanol to remove impurity before drying in an oven at 140 °C for 1.5 h to obtain a superhydrophobic fabric.

### 2.5. Characterization

#### 2.5.1. Contact Angle and Rolling Angle

Contact angle test: The contact angle is tested and characterized by using a DSA100 contact angle tester made by Krüss Company (Hamburg, Germany). The single water output is 6 L of deionized water. After the droplets are completely still, the contact angle is measured five times at different positions on the surface of the sample to be measured, and the average value is taken as the measurement result. 

Rolling angle test: Use the DSA100 contact angle tester made by the German Krüss Company to test and characterize the rolling angle. The single water output is 10 µL of deionized water, which drops on the surface of the sample to be measured. By adjusting the inclination angle of the inclined table, the liquid drops roll completely. The inclination angle at this time is used as the rolling angle and measured five times at different positions on the surface of the sample to be measured, and the average value is taken as the measurement result.

#### 2.5.2. Surface Morphology and Elemental Analysis

The sample to be measured was cut into about 4 cm× 4 cm, fixed on the electron microscope stage by conductive adhesive, sprayed with gold for 60 s, and the surface structure and morphology of nylon 56 fiber and cotton fiber were observed by scanning electron microscope (Hitachi S-4800).

The energy dispersive spectrometer (EDS) attached to Hitachi TM3030 SEM (Tokyo, Japan) was used to scan and analyze the sample to be tested in a vacuum, and the element composition and element content of nylon 56 fiber and cotton fiber surface were determined.

#### 2.5.3. X-ray Photoelectron Spectroscopy Test

X-ray photoelectron spectroscopy (XPS) of nylon 56 fiber and cotton fiber was measured by Thermo Fisher instrument (NEXSA, Phoenix, AZ, USA) and AlKαX-ray source (1486.6 eV).

#### 2.5.4. Acid Resistance Test

Prepare 7 cups of solutions with pH values of 1, 3, 5, 7, 9, 11, and 13 respectively. Seven pieces of superhydrophobic fabrics with a size of 3 cm × 3 cm were cut out from the sample of superhydrophobic fabrics, which were placed in solutions with different pH values for 24 h, and then dried in an oven at 60 °C. Finally, the contact angle and rolling angle of the fabrics were measured.

#### 2.5.5. Friction Fastness Test

A total of 6 pieces of 5 cm × 5 cm superhydrophobic fabric samples were placed on 1000 Cw sandpaper, and the fabric was pressed with a weight of 100 g, and 15 cm was pulled along the scale at a speed of 2 cm/s. The changes of contact angle and rolling angle of the fabric samples rubbed 0, 5, 10, 15, 20, and 25 times respectively were recorded.

#### 2.5.6. Ultraviolet Fastness Test

Five superhydrophobic fabric samples of 5 cm × 5 cm were exposed to ultraviolet light, 15 cm away from the light source, and the contact angle and rolling angle changes of the fabric samples irradiated for 0, 4, 8, 12, and 16 h were recorded, respectively.

#### 2.5.7. Testing of Fastness to Organic Solvents

Seven pieces of 5 cm × 5 cm superhydrophobic fabric samples were soaked in acetone solvent, and the contact angle and rolling angle changes of the fabric samples soaked for 0, 4, 8, 12, 16, 20, and 24 h were recorded, respectively.

#### 2.5.8. Soap Fastness Test

According to AATCC test method 61–2003 test No. 1A, referring to the standard washing machine procedure, seven pieces of 5 cm × 5 cm superhydrophobic fabric samples were washed in the washing machine, and the changes of contact angle and rolling angle of the fabric samples washed for 0, 45, 90, 135, 180, 225, and 270 min were recorded respectively.

#### 2.5.9. Self-Cleaning and Oil–Water Separation Test

Self-cleaning: The original fabric and superhydrophobic fabric are pasted on the surface of the glass slide, placed in a culture dish at an inclined angle, reactive blue 4 dye is added to the surface of the fabric, deionized water is dripped through a dropper to wash the surface of the fabric, and the experimental phenomenon is recorded by taking pictures.

Oil–water separation: In the oil–water separation test, methylene chloride is dyed red with oil red O, and water is dyed blue with methyl blue. Pour the oil/water mixture (the volume of oil and water are both 100 mL) into the oil–water separation device.

## 3. Results and Discussion

### 3.1. Brief Description of the Formation Mechanism of Superhydrophobic Fabric

**Figure 1:**
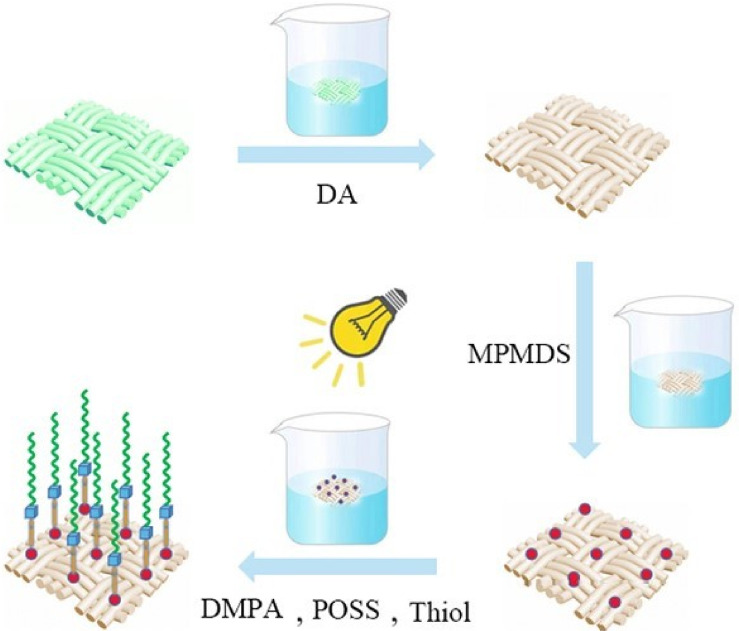
Formation mechanism of superhydrophobic textiles.

First, dopamine molecules oxidize and self-polymerize, resulting in the formation of polydopamine aggregates. In the solution, these aggregates undergo Brownian motion. When they approach a suitable position, they use the interaction force between the polydopamine and fiber substrate as a driving force to adhere to the fiber. On the surface, polydopamine aggregates containing multiple hydroxyl functional groups are formed. Mercaptopropylmethyldimethoxysilane is then hydrolyzed to form silanol, which then condenses with the hydroxyl groups in polydopamine to form hydrogen bonds by introducing mercapto groups on the fiber’s surface. The sulfhydryl group and n-hexadecyl mercaptan on the fiber surface, as well as the double bond in POSS, sequentially undergo click chemical reactions, introducing low surface energy substances on the fiber surface to form a micro-nano rough structure, and finally, a superhydrophobic fabric. The flow chart of preparing superhydrophobic fabric is shown in the Figure 1.

### 3.2. Surface Morphology and Composition of Superhydrophobic Fabrics

**Figure 2:**
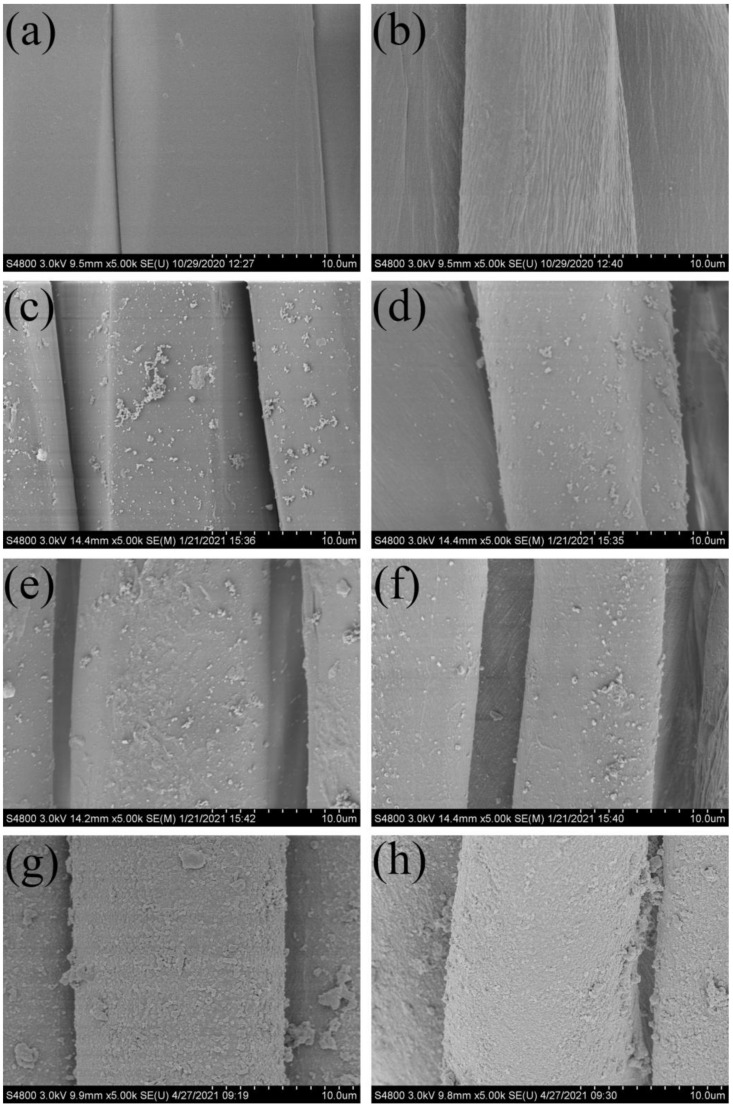
SEM images of (**a**) original nylon 56 fiber, (**b**) original cotton fiber, (**c**) dopamine-modified nylon 56 fiber, (**d**) dopamine-modified cotton fiber, (**e**) sulfhydryl-modified nylon 56 fiber, (**f**) sulfhydryl-modified cotton fiber, (**g**) superhydrophobic nylon 56 fiber, and (**h**) superhydrophobic cotton fiber.

SEM images of the original fabric, dopamine-modified fabric, sulfhydryl-modified fabric, and superhydrophobic fabric are shown in Figure 2. The original nylon 56 fiber surface is smooth and free of impurities, as shown in Figure 2a,b, whereas the original cotton fiber is inherent. There are no other substances attached to the ravine’s form. Following dopamine modification treatment (Figure 2c,d), nylon 56 fibers and cotton fibers are polydopamine-coated to form micro-nano aggregates and a secondary reaction platform. A dense film coating was formed on the surface of nylon 56 fibers and cotton fibers after they were modified with mercaptopropylmethyldimethoxysilane. The mercaptopropylmethyldimethoxysilane SEM images are shown in Figure 2e,f; the hydrolysate adheres uniformly to the fibers. The surface of nylon 56 fibers and cotton fibers significantly changed after the click chemistry reaction, forming tightly arranged nanoparticles and developing a highly hydrophobic rough structure (Figure 2g,h), thus providing the fabric with superhydrophobic properties.

**Figure 3:**
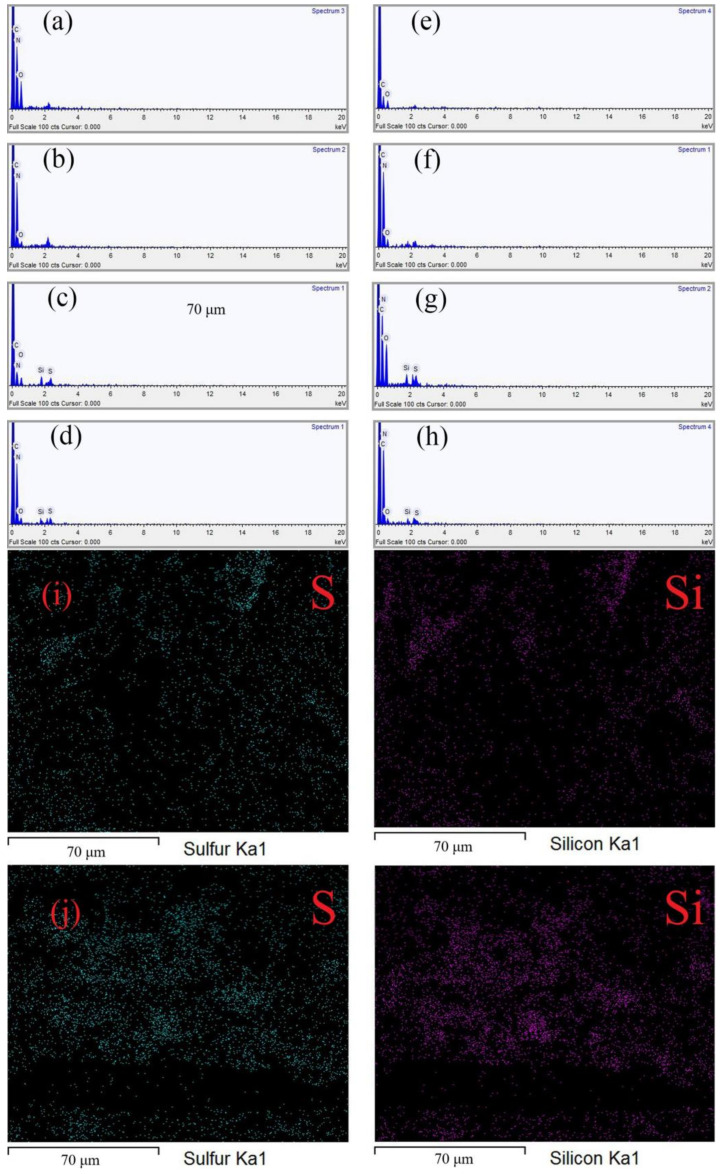
EDS spectrum of (**a**) original nylon 56 fiber, (**b**) dopamine-modified nylon 56 fiber, (**c**) sulfhydryl-modified nylon 56 fiber, (**d**) superhydrophobic nylon 56 fiber, (**e**) original cotton fiber, (**f**) dopamine-modified cotton fiber, (**g**) sulfhydryl-modified cotton fiber, and (**h**) superhydrophobic cotton fiber. (**i**) Element mapping of superhydrophobic nylon-56 fiber, (**j**) element mapping of superhydrophobic cotton fiber.

Figure 3 shows the EDS energy spectrum of nylon 56 fiber and cotton fiber before and after finishing, respectively. Table 1 lists the element contents. Table 1 compares the C, O, and N element content ratio on the dopamine-modified nylon 56 fiber to that in Table 1, which has significantly changed. The presence of N in cotton fiber in Table 1 indicates that polydopamine has been successfully coated on the fiber surface. The appearance of S and Si in Table 1 demonstrates that mercaptosilane successfully hydrolyzes and adheres to the fabric. Furthermore, the S and Si content of nylon 56 fiber and cotton fiber has changed (Table 1). The click chemical reaction has been successful, indicating that POSS and mercaptans have been grafted to the fiber, thus showing super-hydrophobicity on the fabric surface. Figure 3i,j shows the distribution of S and Si on the surface of the modified fabric; the elements were evenly distributed.

**Figure 4:**
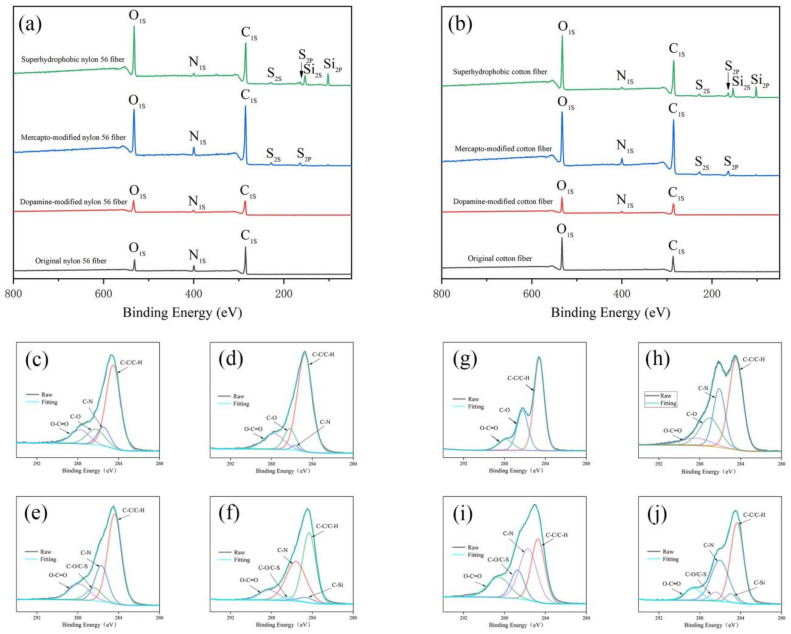
(**a**) XPS spectra of the original nylon-56 fiber, dopamine-modified nylon-56 fiber, sulfhydryl-modified nylon 56 fiber, and superhydrophobic nylon-56 fiber; (**b**) XPS spectra of raw cotton fiber, dopamine-modified cotton fiber, sulfhydryl-modified cotton fiber and superhydrophobic cotton fiber. High-resolution C1s spectrum of original nylon-56 fiber (**c**), dopamine-modified nylon-56 fiber (**d**), sulfhydryl-modified nylon 56 fiber (**e**), superhydrophobic nylon-56 fiber (**f**); raw cotton fiber (**g**), dopamine-modified cotton fiber (**h**), sulfhydryl-modified cotton fiber (**i**), superhydrophobic cotton fiber (**j**).

To analyze the changes in the chemical composition of the fabric surface during the entire reaction, we performed X-ray photoelectron spectroscopy (XPS). As shown in Figure 4a,b, for the original nylon 56 fabric, the spectrum shows that it contains C, O, and N peaks. For the original cotton fabric, the spectrum shows that it contains C and O peaks. For the superhydrophobic nylon 56 fiber and cotton fiber, we can clearly observe that four new peaks appear, namely 150.5 eV (Si 2s), 100.1 eV (Si 2p), 229.0 eV (S 2s), and 164.0 eV (S 2p), which shows that the low surface energy substance is successfully grafted on the fiber surface through click coupling. The C1s peaks of the original nylon 56 fabric showed four different peaks around 284.6 eV, 285.7 eV, 286.5 eV, and 288.2 eV corresponding to the C-C/C-H bond, C-N bond, C-O bond, and O-C=O bond (Figure 4c). The C1s peak of the cotton fabric appears at three different peaks around 284.6 eV, 286.5 eV, and 288.2 eV, corresponding to the C-C/C-H bond, C-O bond, and O-C=O bond (Figure 4g). Dopamine forms a polydopamine coating on the fiber surface. So a C-N bond (285.7 eV) appeared (Figure 4d,h), followed by a click reaction after the mercaptosilane was hydrolyzed, POSS and mercaptan were introduced into the fiber, and finally a C-S bond (286.5 eV) and C-Si bond (284.9 eV) (Figure 4e,f,i,j). The above characteristic peaks indicate that thiol and POSS were successfully grafted onto the polydopamine coating by thiol–ene click chemistry [12].

### 3.3. The Effect of Reactant Ratio on the Superhydrophobic Finishing of Fabrics

**Figure 5:**
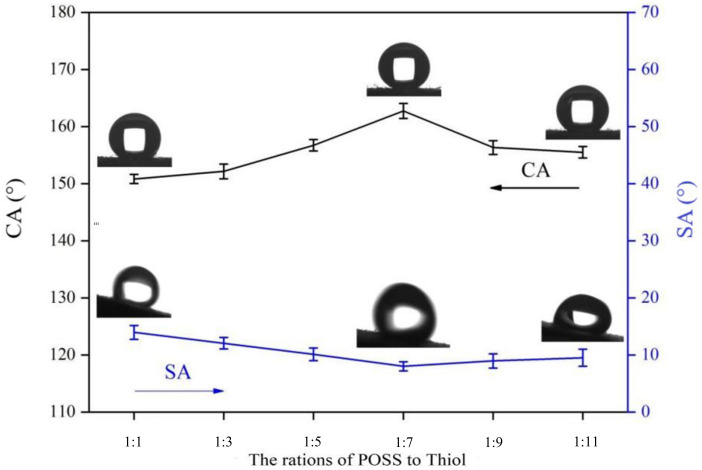
Changes in CA and SA with the ratio of octavinyl-POSS to cetyl mercaptan.

Here, octavinyl-POSS was used as a bridge to graft mercaptosilane and thiol to generate POSS-based polymers on the fiber surface in turn using the thiol–ene click reaction. POSS monomer and mercaptan are both highly hydrophobic reagents, and the amount ratio between them has a significant impact on the wettability of the fiber surface. Figure 5 shows the relationship between CA and SA and the POSS/thiol ratio. The CA reaches 162°, the SA reaches 8°, and the superhydrophobic performance is the best when the molar mass ratio of POSS to mercaptan is 1:7. This is because the mercaptosilane has occupied a double bond, leaving seven. One double bond grafting point is reserved for thiols. The sulfhydryl groups and double bonds are consumed one by one. It embodies the click chemistry modular properties. The CA did not increase because the amount of mercaptan increased, possibly because the mercaptan had reached a saturated state on the fiber. The results show that the best superhydrophobic performance is achieved when the molar mass ratio of POSS to mercaptan is 1:7.

### 3.4. Mechanical Stability of the Superhydrophobic Fabric

**Figure 6:**
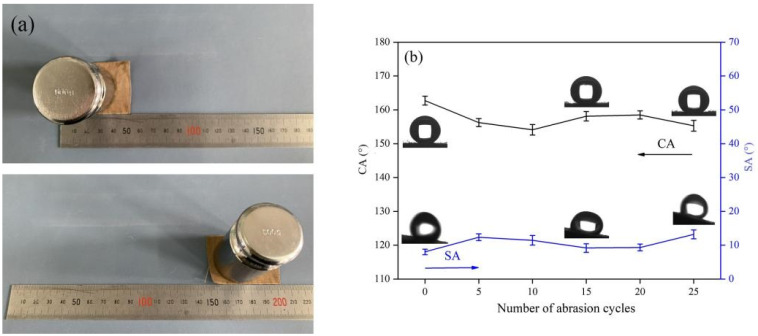
(**a**) Picture of sandpaper abrasion test. (**b**) Changes in CA and SA of superhydrophobic fabrics after 25 abrasion cycles.

Considering that the mechanical stability of superhydrophobic fabrics is a crucial factor in practical application, we quantitatively analyze the mechanical durability of superhydrophobic fabrics by abrasive paper wear test (Figure 6a), and the change of wettability is shown in Figure 3, Figure 4, Figure 5 and Figure 6. With the increase in wear times, the CA of the fabric can still be kept above 150, which is because the surface of the generated POSS-based polymer has a strong inorganic core and is cross-linked with the fabric substrate through firm chemical bonds, so it is not easy to be damaged and shed by external forces. Excellent mechanical durability promotes the application potential of superhydrophobic textiles.

### 3.5. Chemical Stability and Durability of Superhydrophobic Fabrics

**Figure 7:**
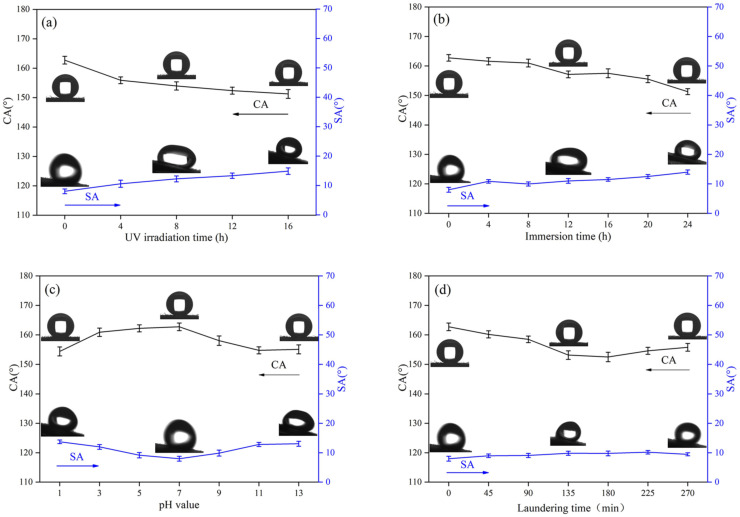
The changes in the CA and SA of superhydrophobic nylon-56/cotton-interwoven fabric under the following conditions: (**a**) UV irradiation over various times; (**b**) soaking in acetone over various times; (**c**) soaking in various pH solutions over 48 h; (**d**) washing over various times.

In the process of daily use, fabrics inevitably come in contact with complex environments. Therefore, industrial production requires a relatively high chemical stability and durability of superhydrophobic fabrics. To evaluate the effect of UV radiation on superhydrophobic fabrics, fabric samples were exposed to UV light. After 16 h of UV irradiation, the CA of the fabric significantly decreased (Figure 7a). However, the superhydrophobic performance was still maintained, demonstrating that the superhydrophobic fabric has excellent resistance to strong light. To test the effect of organic solvents on the fabric, the samples were soaked in acetone for a certain time period, then washed with deionized water, and dried at 60 °C. As shown in Figure 7b, after the fabric is soaked for 24 h, the CA still remains at >150°. This may be because the POSS-based polymer on the fiber surface has good resistance to organic solvents, indicating the fabric’s excellent solvent resistance. Figure 7c shows the CA and SA after immersing the superhydrophobic fabric in solutions with different pH values (pH = 1, 3, 5, 7, 9, 11, and 13) for 48 h. Compared with strong alkaline liquids, the results show that the fabric has better resistance to strong acidic liquids. Although it has a certain impact on the hydrophobic properties of the fabric, the effect is less. This may be because the air trapped on the fabric’s surface can inhibit acid and alkali corrosion. According to AATCC test method 61–2003 test No. 1A, the fabric washing durability is tested with reference to the standard washing machine procedure. The washing time is 0, 45, 90, 135, 180, 225, and 270 min. As shown in Figure 7d, the effect of washing on the CA and SA of the fabric is not great, and the fabric still retains excellent water repellency. This may be attributed to the super adhesion of dopamine, which makes it difficult for POSS-based low surface energy substances to fall off the fiber surface. Therefore, the superhydrophobic fabric manufactured by this method has excellent UV resistance, solvent resistance, acid and alkali resistance, and water washing resistance is expected to be mass-produced.

### 3.6. Self-Cleaning Performance of the Superhydrophobic Fabric

**Figure 8:**
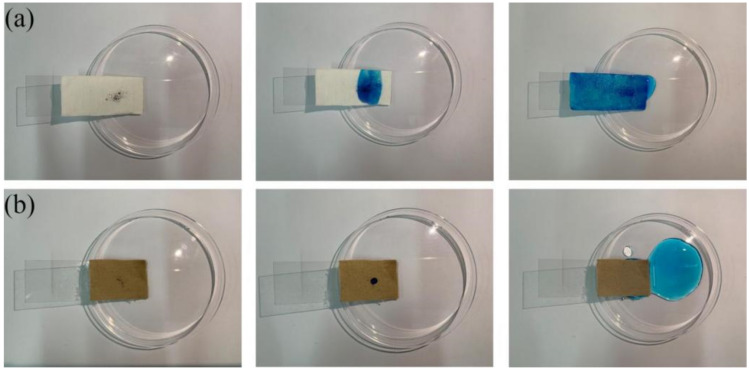
Self-cleaning test of fabric: (**a**) original fabric and (**b**) superhydrophobic fabric.

**Figure 9:**
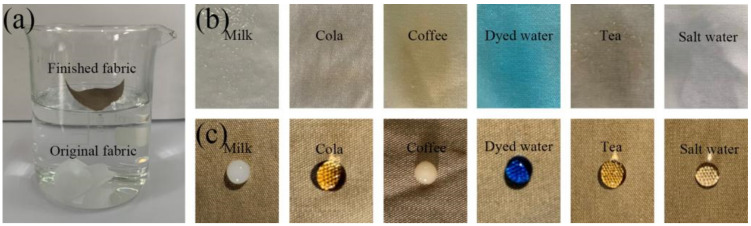
(**a**) The water soaking phenomenon of the original fabric and the superhydrophobic fabric, (**b**) the state of different droplets on the original fabric, and (**c**) the state of different droplets on the superhydrophobic fabric.

Superhydrophobic fabrics should have anti-fouling and self-cleaning properties. When contaminants contact the fabric’s surface, they should be easy to remove by washing them with water. As shown in Figure 8a,b, the blue dye did not move with the water droplets for the original fabric but spread to the entire fabric. However, the contaminants on the superhydrophobic fabric were easily taken away by the water droplets, thus leaving a clean surface. To confirm the water repellency of the superhydrophobic fabric, the original fabric and the superhydrophobic fabric were placed in water. The results demonstrated that the original fabric sank into the water while the superhydrophobic fabric was suspended on the water surface after being released (Figure 9a). Moreover, we conducted anti-fouling tests using saltwater, coffee, milk, dyed water, cola, and tea to explore the practicality of coated fabrics in daily life. As shown in Figure 9b,c, the original fabric was completely wetted by contamination; however, the droplets on the surface of the superhydrophobic fabric were spherical. These results confirm that the modified fabric exhibits excellent self-cleaning and anti-fouling properties.

### 3.7. Oil–Water Separation Performance of the Superhydrophobic Fabric

**Figure 10:**
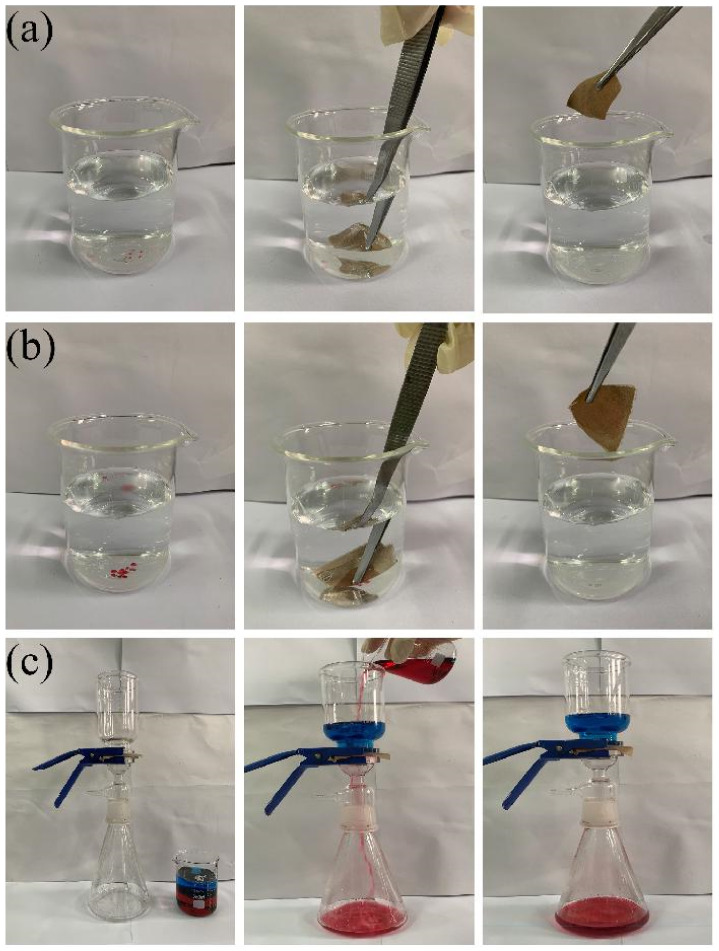
(**a**,**b**) The selective adsorption of superhydrophobic fabric to methylene chloride (dyed with oil red O) and carbon tetrachloride (dyed with oil red O) in water, (**c**) the oil–water separation test of superhydrophobic fabric.

Superhydrophobic fabrics are extensively used in industrial production to treat oil–water mixtures. Here, we investigated the oil–water separation performance of superhydrophobic fabrics. Methylene chloride and carbon tetrachloride were dyed red with oil red O, as shown in Figure 10a,b. The superhydrophobic fabric completely adsorbed the red-colored solvent, demonstrating its ability to adsorb oil stains. A schematic of the effect of using a superhydrophobic fabric for an oil–water separation test is shown in Figure 10c. The fabric (6 cm × 6 cm) is sandwiched between the oil–water separation device, and the methylene chloride is labeled with oil red O as red, and the water with methyl blue as blue. Pour the oil/water mixture (both the oil and water volumes are 100 mL) onto the superhydrophobic fabric; the red oil completely penetrates the fabric, and the blue water is trapped on the fabric. Consequently, the prepared superhydrophobic fabric has excellent oil–water separation properties and can be used in sewage treatment.

## 4. Conclusions

In this study, we synthesized a superhydrophobic fabric by coating polydopamine on the fiber surface, introducing abundant hydroxyl functional groups, and providing reaction points for grafting sulfhydryl functional groups before grafting POSS and mercaptans via thiol–ene click chemistry. The base polymer provides a low surface energy substance for the fiber surface and improves the fabric’s surface roughness, resulting in a superhydrophobic fabric. The CA is 162°, whereas the SA is 8°. The superhydrophobic fabrics have high mechanical stability, can withstand mechanical abrasion, UV radiation, organic solvents, water washing, acid–base environments, and their excellent self-cleaning and oil–water separation properties are adequate for various applications.

## Figures and Tables

**Table 1:** Element composition content of (a) original nylon 56 fiber, (b) dopamine-modified nylon 56 fiber, (c) sulfhydryl-modified nylon 56 fiber, (d) superhydrophobic nylon 56 fiber, (e) original cotton fiber, (f) dopamine-modified cotton fiber, (g) sulfhydryl-modified cotton fiber, and (h) superhydrophobic cotton fiber.

Element	C	O	N	S	Si	Total
(a) (Weight %)	44.732	40.314	14.954	0	0	100
(b) (Weight %)	56.618	15.423	27.959	0	0	100
(c) (Weight %)	47.540	29.679	15.605	2.738	4.439	100
(d) (Weight %)	63.194	14.507	18.899	2.293	1.108	100
(e) (Weight %)	50.925	49.075	0	0	0	100
(f) (Weight %)	71.872	21.448	6.680	0	0	100
(g) (Weight %)	46.028	41.042	9.946	1.305	1.679	100
(h) (Weight %)	63.325	12.012	23.366	0.487	0.810	100

## Data Availability

The data presented in this study are available on request from the corresponding author.
